# Uncovering a reconstructive solid–solid phase transition in a metal–organic framework

**DOI:** 10.1098/rsos.171355

**Published:** 2017-11-29

**Authors:** L. Longley, N. Li, F. Wei, T. D. Bennett

**Affiliations:** 1 Department of Materials Science and Metallurgy, University of Cambridge, 27 Charles Babbage Road, Cambridge CB3 0FS, UK; 2 State Key Laboratory of Silicate Materials for Architectures, Wuhan University of Technology, Hubei 430070, People's Republic of China

**Keywords:** metal–organic framework, phase transition, X-ray, porous material

## Abstract

A nanoporous three-dimensional metal–organic framework (MOF), ZnPurBr undergoes a transition to a previously unreported high-temperature phase, ZnPurBr-ht. The transition, which proceeds without mass loss, is uncovered through the use of differential scanning calorimetry (DSC). The new crystal structure was solved using single-crystal X-ray diffraction, and the mechanical properties of both phases investigated by nanoindentation and density functional theory. The anisotropy of the calculated Young's moduli showed good agreement with the crystallographic alignment of the stiff purinate organic linker. The results provide a prototypical example of the importance of the use of DSC in the MOF field, where its use is not currently standard in characterization.

## 1. Introduction

Metal–organic frameworks (MOFs) are a subclass of coordination polymers, and are structures formed from the bonding of organic ligands to inorganic secondary building units (SBUs) in infinite arrays, with the connectivity and topology of the structure being determined by the geometry of the organic linker and the SBU [1]. Zeolitic imidazolate frameworks (ZIFs) are a subset of MOFs with the general formula M(Im)_2_, where M is a divalent metal cation, typically Zn^2+^ or Co^2+^, and Im^−^ is an imidazolate derivative [2]. The high porosity and chemical tunability of ZIFs have made them attractive for many potential applications, especially those involving carbon capture, gas adsorption and separation, catalysis and sensing [3,4]. ZIFs have been reported as having superior thermal and chemical stabilities relative to other classes of MOFs, which makes them particularly attractive for industrial applications [5].

Given the various stresses imposed upon them in their proposed applications, the study of the thermo-mechanical stability of ZIFs is vital if they are to come to industrial fruition [6]. Thermogravimetric analysis (TGA) is frequently used to identify the limits of MOF thermal stability [7]. However, there are two major drawbacks with this widely used technique: (i) it is unable to identify those transitions which do not involve mass loss, and (ii) framework decomposition temperatures are heavily dependent upon atmospheric conditions. Despite phase change phenomena having such a profound effect on the chemical properties of ZIFs, very few reports exist due to the lack of differential scanning calorimetry (DSC) characterization of MOFs in the literature.

The reported instances have demonstrated that ZIFs containing bidentate ligands are not as thermally stable as may appear from TGA traces alone. Responses to temperature, pressure or shear stress in the family have ranged from phase transitions, to collapse, or, as recently shown, melting [8–11]. For example, ZIF-7 [Zn(bIm)_2_] (bIm = benzimidazolate, C_5_H_7_N_2_^−^), which adopts the same network topology as sodalite, is observed to undergo reversible phase change behaviour to more open framework structures upon absorption/desorption of guest molecules and heating [10]. On the other hand, ZIF-4 [Zn(Im)_2_] (Im = imidazolate, C_3_H_3_N_2_^−^), which adopts the same network topology as the mineral variscite, CaGa_2_O_4_, exhibits an irreversible reconstructive phase change upon heating to a more dense phase, via an amorphous silica-like intermediate [12].

Such changes may enhance the industrial functionality of various MOF structures, as has been observed for a variety of gate opening and breathing MOFs [13,14]. However, many lead to undesirable outcomes, and thus methods to improve the thermal stability of MOFs are highly sought after. One possible factor is the connectivity of the organic ligand, and, at the time of writing, no instances of the full thermo-mechanical characterization of a tridentate ligand-containing MOF [15,16] had yet been attempted. Our motivation for this work was thus to probe whether inclusion of a tridentate ligand would enhance the thermal and mechanical stability of a ZIF, by probing its behaviour with DSC and nanoindentation.

**Figure 1.**
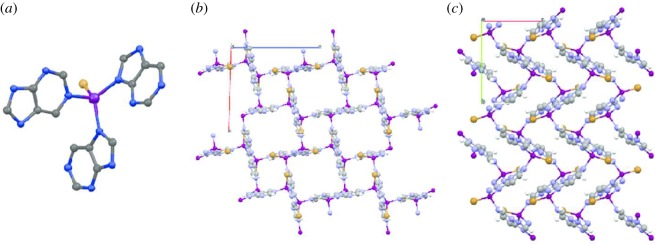
(*a*) Local zinc coordination environment in ZnPurBr. (*b*) Unit cell of ZnPurBr. (*c*) Unit cell of ZnPurBr-ht. C – grey, N – blue, H – white, Zn – purple, Br – orange.

A suitable example to study was found in the ZnPurBr framework, first synthesized by Wright and co-workers [17], which contains tetrahedral Zn^2+^ centres linked by three purinate (pur—C_5_H_4_N_4_^−^) ligands. Two of these bond through the NCN moiety of the imidazolate section of the purinate ligand, as in other ZIFs. The third arises as a result of the electron donating N atom on the opposite side of the ligand. Unusually, the coordinative demands of Zn^2+^ are satisfied by a monodentate Br (figure 1*a*). ZnPurBr crystallizes in the space group *P*2_1_/n, with *a* = 13.636 Å, *b* = 10.209 Å, *c* = 14.124 Å, *β* = 91.568° and *V* = 1965 Å^3^, and with a solvent accessible volume (SAV) of *ca* 25%, using a probe radius of 1.2 Å (figure 1*b*), though pore-templating *N*,*N*-dimethylformamide molecules occupy the cavities and prevent ingress of any further guests.

## 2. Results and discussion

**Figure 2.**
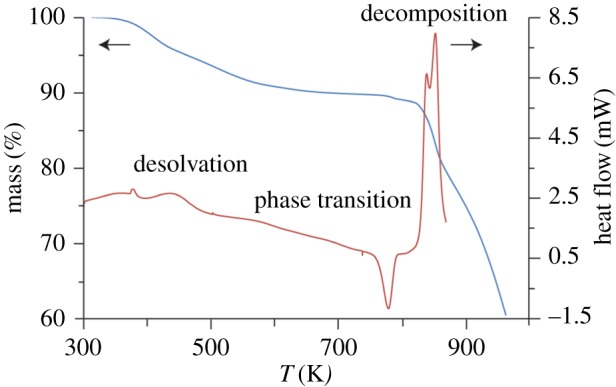
DSC (red) and TGA (blue) of ZnPurBr. The measurements were conducted at a ramp rate of 10 K min^−1^. Desolvation can be seen as an endotherm in the DSC at 400 K concurrent with a mass loss of 9.967% in the TGA. An exotherm is observed at 762 K with no concurrent mass loss.

A TGA trace of the as-synthesized ZnPurBr (figure 2) showed a mass loss associated with thermal decomposition above 800 K, in a manner fully consistent with the literature. A mass loss of 9.97% at 400 K was also recorded, in good agreement with the reported framework-templating DMF content [17], and concurrent with an endotherm in the DSC at 400 K. Curiously, however, an exothermic process with no accompanying mass loss in the TGA was also observed at 762 K, before the endotherm associated with thermal decomposition.

**Figure 3.**
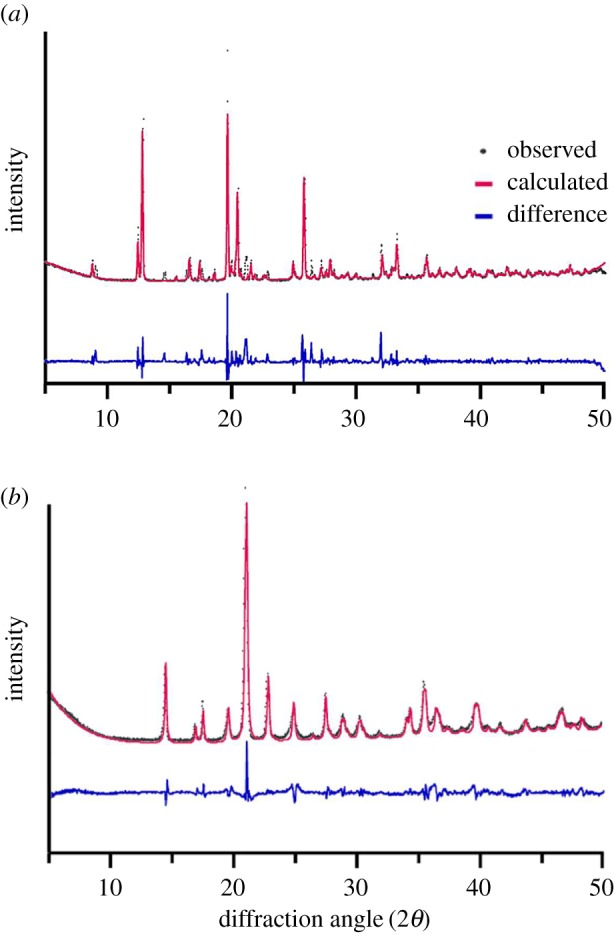
Pawley refinements of (*a*) ZnPurBr and (*b*) ZnPurBr-ht at 298 K.

In order to identify this feature, a sample of ZnPurBr was heated to 804 K, i.e. just above the exotherm, followed by a return to room temperature. Powder X-ray diffraction (PXRD) carried out confirmed an irreversible phase transition had taken place (figure 3), to a previously unknown phase. Single crystals were examined, and retained the prism-like morphology of the original sample, though extensive cracking was observed. Such cracking is indicative of reconstructive phase transitions in ZIFs, having been witnessed in a pressure-induced transition of ZIF-zni [18].

The crystal structure of the high-temperature phase was then determined using single-crystal X-ray crystallography. ZnPurBr-ht crystallizes in an orthorhombic space group *P*2_1_2_1_2_1_ with *a* = 7.592(7) Å, *b* = 10.073(5) Å, *c* = 10.104(3) Å and V = 772.7(8) Å^3^ (figure 1*c*). The local environment around the Zn centre was retained. This reduction in density, when adjusted for the number of tetrahedral atoms per volume, is over 20% and explains the absence of any SAV in the high-temperature phase. Pawley fitting of the high-temperature structural file to the powder diffraction pattern gained yielded good agreement. The topology of this new phase was determined to be *srs*, and no features in the DSC or TGA were observed until 823 K, where decomposition took place (electronic supplementary material, figure S1).

To probe the mechanism of the phase transformation, the detail of the purinate linkers was removed in order to visualize the Zn^2+^ connectivity of the ZnPurBr and ZnPurBr-ht structures (electronic supplementary material, figure S2), following methodology used previously to identify the mechanism of pressure-induced transformations of ZIF-zni [18]. Although some structural features appear to recur in both ZnPurBr and ZnPurBr-ht, namely the kinked chains and the six-membered rings, the complexity of the crystalline structure and the bonding-defied assignment of a plausible mechanism. The high temperature of transformation is indicative of a reconstructive transformation, though in this instance, unlike others [19], no amorphous intermediates were observed.

**Figure 4.**
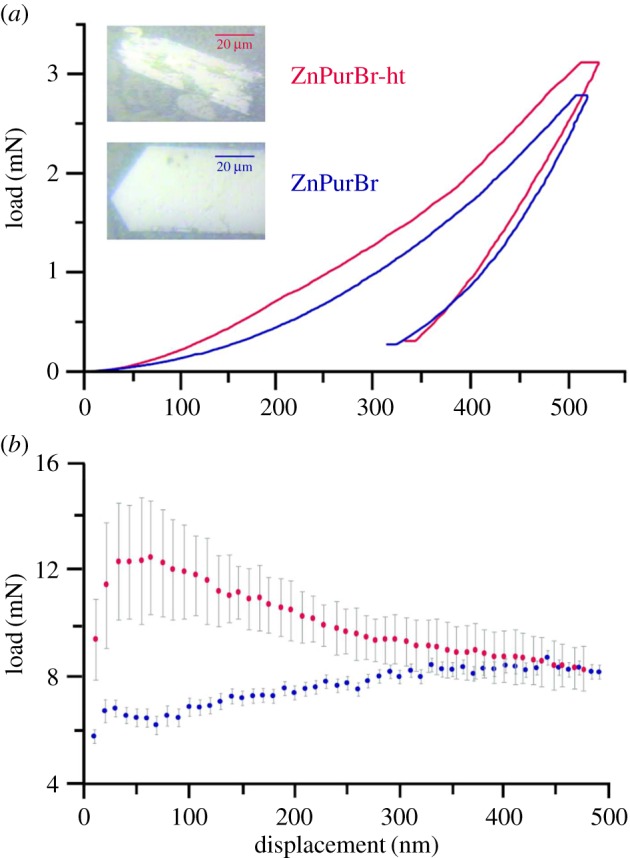
(*a*) Load versus displacement plots for ZnPurBr (blue) and ZnPurBr-ht (red), with accompanying optical images. (*b*) Average displacement versus *E* for both samples. Data collected in the first 100 nm were not included in the calculations due to surface effects.

Young's moduli, *E,* of the two frameworks were evaluated by nanoindentation [20]. While anisotropy in the mechanical response of MOF crystals is established, the prism-like crystal morphologies here dictated that only the vicinity of the [011] direction could be indented. The two samples possess characteristic load–displacement curves (figure 4*a*), and the stiffness as measured from the load displacement data remained approximately constant after the initial 100 nm, allowing a single value of *E* to be identified for each framework (figure 4*b*). As expected given its higher density relative to the ambient temperature phase (2.27 and 1.91 g cm^−3^, respectively), ZnPurBr-ht supports a higher load and is accordingly stiffer along this direction (9.3 GPa cf. 7.8 GPa). The two displayed similar elastic recoveries and creep deformation. The higher scatter in the measurements of ZnPurBr-ht relative to ZnPurBr is attributed to the heavily fractured nature of the indented crystal (figure 4*a inset*), which results from the single-crystal phase transformation.

Density functional theory (DFT) was carried out to further investigate the change in mechanical properties upon the thermal phase transition. Considerable anisotropy was found in both frameworks, with *E*_min_ and *E*_max_ values for ZnPurBr of 16.7 and 174 GPa and corresponding values for ZnPurBr-ht of 41.5 and 174 GPa. Reuss averaging values for the room- and high-temperature phases were 48.3 and 95.6 GPa. While quantitative agreement between the experimental and computationally derived values was poor, similar disparities have been reported in other hybrid frameworks [21]. The significantly worse agreement for the high-temperature phase is ascribed to the severe cracking witnessed upon the phase transition. In a qualitative sense, the increase in stiffness was, however, verified. The DFT modelling also confirmed that the directions of high stiffness correspond to directions along which the purinate linkers are aligned (electronic supplementary material, figures S3 and S4).

**Figure 5.**
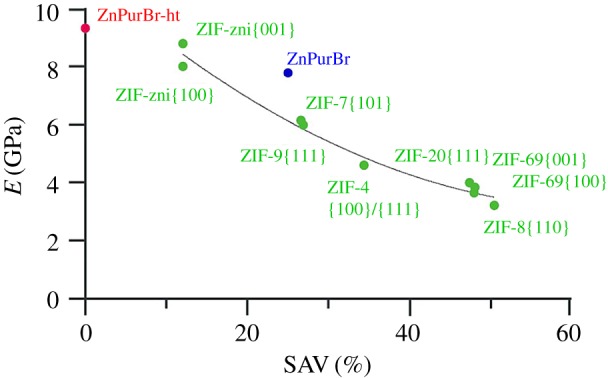
Elastic modulus plotted as a function for SAV for ZIFs reported in the literature [6], along with ZnPurBr and ZnPurBr-ht.

Existing studies on bidentate ligand-containing ZIFs has shown the extent of SAV to have a significant effect on their stiffness (figure 5) [8]. Notably, more open frameworks exhibit a great compliance under the indenter tip, which is promising for the use of MOFs as sensors on microcantilevers, though must be balanced by the mechanical stability. Neither framework studied here follows the trend established by previous studies on bidentate linkers. The ambient temperature structure is less compliant, indicative of a greater degree of metal–ligand–metal connectivity and associated reduction in the degrees of freedom of movement within the structure. This same argument has previously been used to explain the high *E* values found for MOFs in the UiO-family, which consist of [Zr_6_O_4_(OH)_4_] clusters, linked with one another via 12 1,4-benzodicarboxylic acid struts. Comparison with the bidentate purine-containing ZIF-20 partly illustrates the increase in *E*. The high-temperature structure is more compliant than would be expected, though the validity of extrapolating the trend determined from previous study into regions of negligible SAV is questionable.

## 3. Conclusion

A new phase, ZnPurBr-ht, was identified through DSC experiments on ZnPurBr. The mechanical properties of the new phase and the as-synthesized phase were characterized by nanoindentation and DFT, finding large differences. This work represents the first study of the mechanical properties of a tridentate ZIF, and shows the extra connectivity to confer additional framework rigidity. The study is a prototypical example of how the thermal stability of metal–organic frameworks cannot be derived solely from a TGA trace, and that a combination of DSC and/or high-temperature X-ray studies should also be used.

## 4. Material and methods

### 4.1. Synthesis

ZnPurBr was synthesized by the method reported by Kahr *et al.* [17].

### 4.2. X-ray diffraction

Room temperature PXRD data (2*θ* = 5–40°) were collected using a Bruker D8 DAVINCI diffractometer using (λ = 1.540598 Å) with a LynxEye position sensitive detector in Bragg-Brentano parafocusing geometry. Pawley fitting was performed using the routines implemented in the TOPAS-Academic v5 package [6]. Diffraction data for the room temperature sample at 298 K was fitted using the reported cif file for ZnPurBr [17]. A Pawley fit was performed, yielding refined cell parameters of *a* = 13.6030 Å, *b* = 10.0679 Å, *c* = 14.0324 Å (*R*_wp_ = 18.85%). Data for the recovered high-temperature phase were fitted using the experimental cif file for ZnPurBr-ht (below). A Pawley fit was performed, yielding refined cell parameters of *a* = 7.5863 Å, *b* = 10.0271 Å, *c* = 10.1114 Å (*R*_wp_ = 9.81%).

Single-crystal X-ray diffraction was performed using an Oxford Diffraction Gemini A Ultra X-ray diffractometer with Mo-Ka radiation (λ = 0.71073 Å) operated at 50 kV and 40 mA. Data were collected at 120 K under nitrogen flow. Data collection, pre-analysis and reduction were done using the CrysAlisPro software from Agilent Technologies. A face-based absorption correction was applied after the final structure model was achieved. For structure solution, Olex v2 interface was used equipped with the ShelX program using direct methods. The CCDC number for the deposition is 1573648.

### 4.3. Differential scanning calorimetry and thermal gravimetric analysis

Thermal measurements were conducted using a TA instruments Q600 SDT (DSC and TGA) and Q2000 DSC in an inert argon atmosphere. The samples were placed in either a platinum (Q2000 DSC) or alumina (Q600 SDT) crucibles in the DSC/TGA at room temperature and heated at rates of either 10 or 20 K min^−1^ to the specified temperature before being cooled at a natural rate to room temperature.

### 4.4. Nanoindentation

Young's modulus (*E*) of the samples was measured using an MTS Nanoindenter XP at ambient conditions. The Miller indices of single crystals of ZnPurBr were predicted using the Mercury software [22]. Samples were mounted in an epoxy resin and polished using increasingly fine diamond suspensions. Indentation experiments were carried out under the dynamic displacement controlled mode at a constant strain rate of 0.05 s^−1^. All tests were conducted using a three-sided pyramidal (Berkovich) diamond indenter tip to a maximum surface penetration depth of 500 nm. The load–displacement data collected were analysed using the Oliver & Pharr method [20]. A Poisson's ratio of 0.2 was used, in accordance with prior studies on ZIF materials [6].

### 4.5. Computational details

The computational method used was based on DFT, with the Vienna Ab initio Simulation Package (VASP) [23–25]. With VASP, the projector augmented-wave Perdew–Burke–Ernzerhof (PAW-PBE) potential [26] was used within the generalized gradient approximation of the DFT. The following VASP parameters were adopted: (i) a relatively high-energy cut-off of 400 eV; (ii) the use of the Davidson block iteration scheme in the optimization of the wave functions; (iii) an electronic convergence criterion of 10^−5^ eV; (iv) a small tolerance for ionic relaxation with force convergence of 10^−3^ eV Å^−1^ and a Monkhorst–Pack special k-point 5 × 5 × 5 mesh was used for sampling the Brillouin zone.

For the mechanical properties calculations, we used the strain–stress analysis approach [27]. In this approach, a small strain of plus or minus 1% is applied to each independent strain element in the fully relaxed structure. The six stress components are calculated for each strain e_j_ applied to the structure. From the calculated C_ij_ values, one can obtain bulk mechanical properties (bulk modulus *K*, shear modulus *G*, Young's modulus *E* and Poisson's ratio *g*) using the established Voigt–Reuss–Hill scheme [28].

## Supplementary Material

Uncovering A Reconstructive Solid-Solid Phase Transition in a Metal-Organic Framework

## Data accessibility

Data have been made available on the University of Cambridge open data service, with ID numbers of CDDE61C5-1A43-46A9-9080-79EF20881BB1and 8C547D7A-92CD-4930-9B0F-B75FB8655BDE.
