# Supplementary material for: Uncovering a reconstructive solid–solid phase transition in a metal–organic framework
Source: R Soc Open Sci. 2017 Nov 29;4(11):171355. doi: 10.1098/rsos.171355 (PMC5717694; doi:10.1098/rsos.171355)
Supplement: Uncovering A Reconstructive Solid-Solid Phase Transition in a Metal-Organic Framework [file rsos171355supp1.docx]

Uncovering A Reconstructive Solid-Solid Phase Transition in a Metal-Organic Framework

*L. Longley,*^1^ *N. Li,^1,2^ F. Wei^1^ and T. D. Bennett*^1^*

^1^ Department of Materials Science and Metallurgy, University of Cambridge, 27 Charles Babbage Road, Cambridge, CB3 0FS

^2^ State Key Laboratory of Silicate Materials for Architectures, Wuhan University of Technology, Hubei, 430070, China


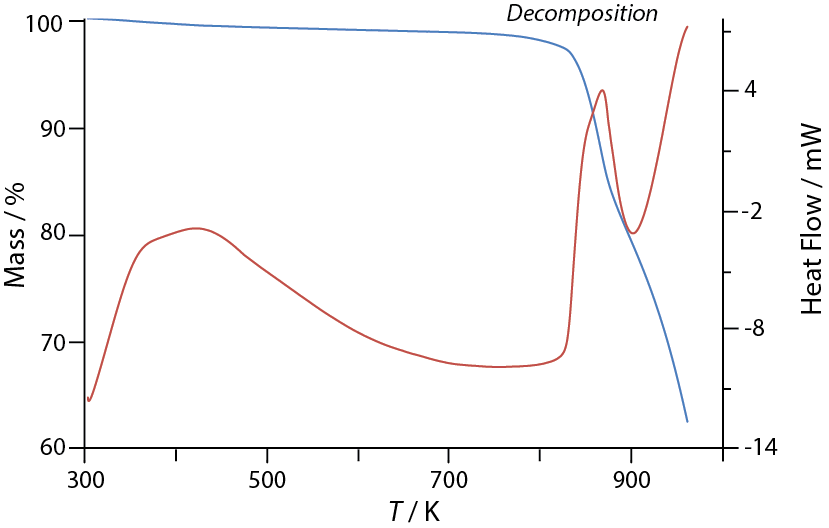


**Figure S1.** DSC (red) and TGA (blue) of ZnPurBr-ht. The measurements were conducted at 20 K/min. The TGA and DSC traces are featureless indicating a thermal stability up to 823 K.


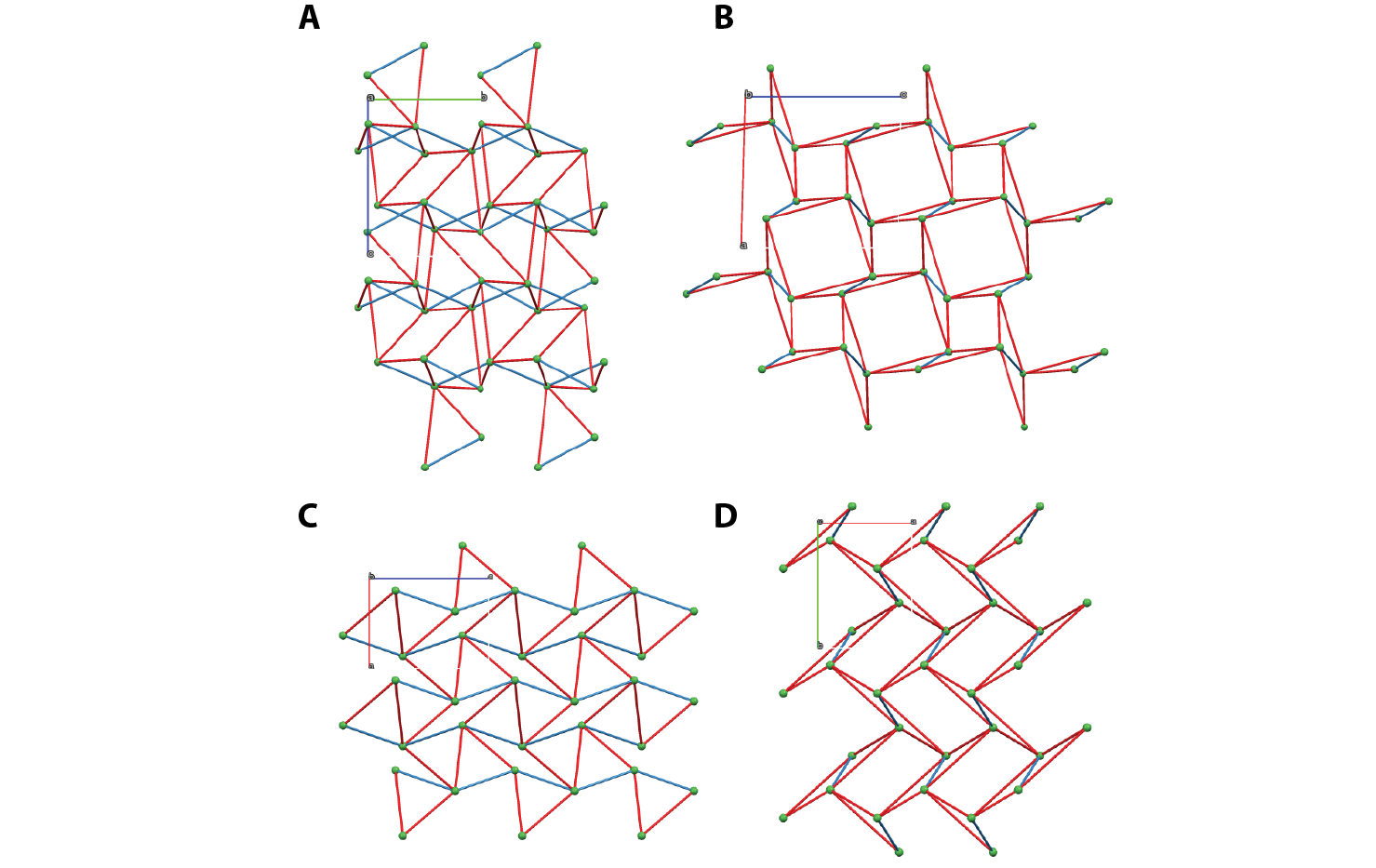


**Figure S2.** Topological projections down the unit cell axes of a) and b) ZnPurBr, and c) and d) ZnPurBr-ht. In order to represent the two different modes of coordinate bonding within the structures, NCN connections within the imidazolate are distinguished from those across the purinate moiety by colour. Trans-purinate bonds: Red, NCN bonds: Blue, Zn centers: Green.


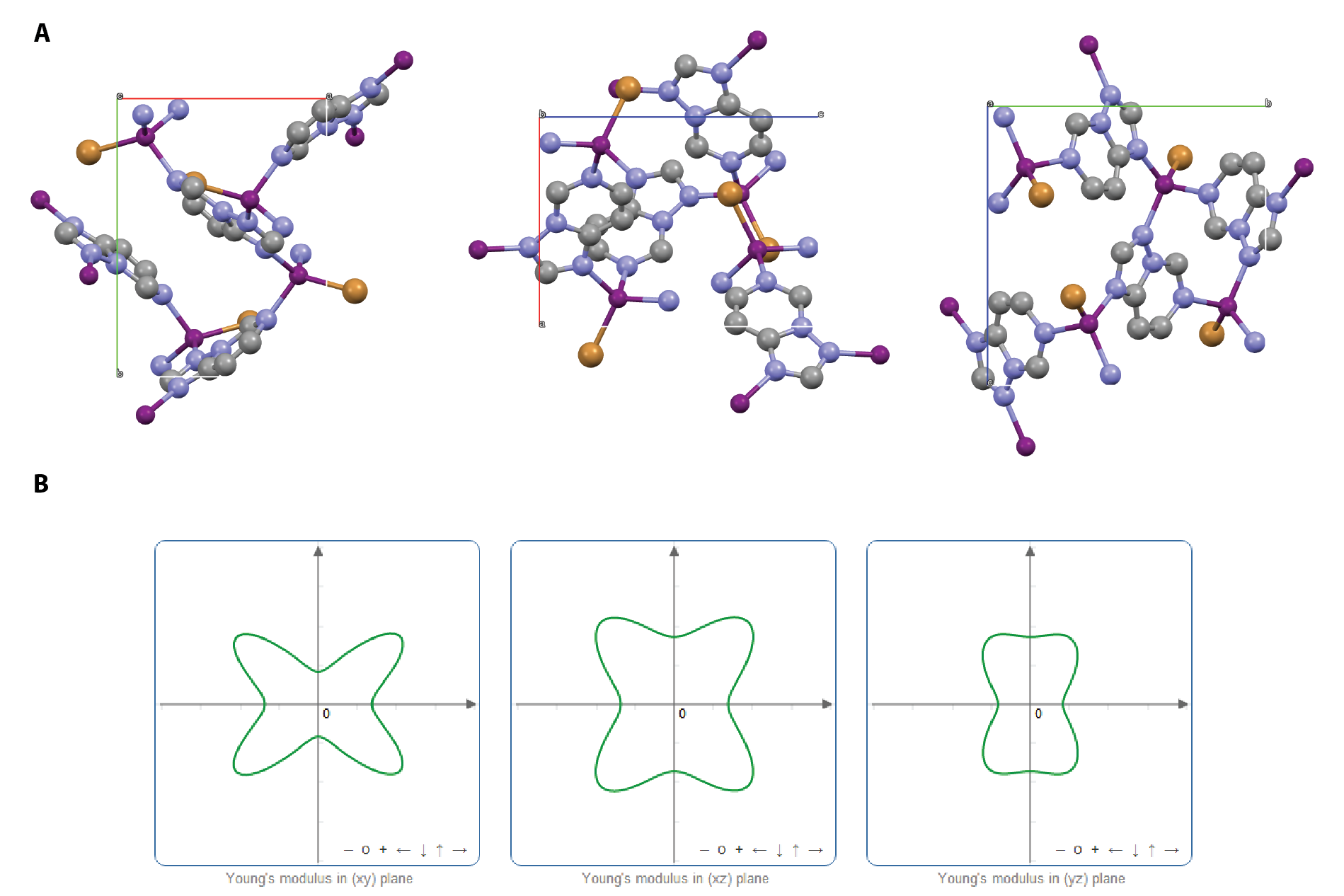


Figure S3. (a) Two dimensional slices through the computationally derived iso-surface of the Young’s Moduli in the ZnPurBr structure. Left: xy plane, center: xz plane, right: yz plane. Bottom: Unit cells of the LT ZnPurBr structure in the corresponding planes. Purple: Zinc, Blue: Nitrogen, Grey: Carbon, Orange: Bromine. Hydrogen atoms omitted for clarity.


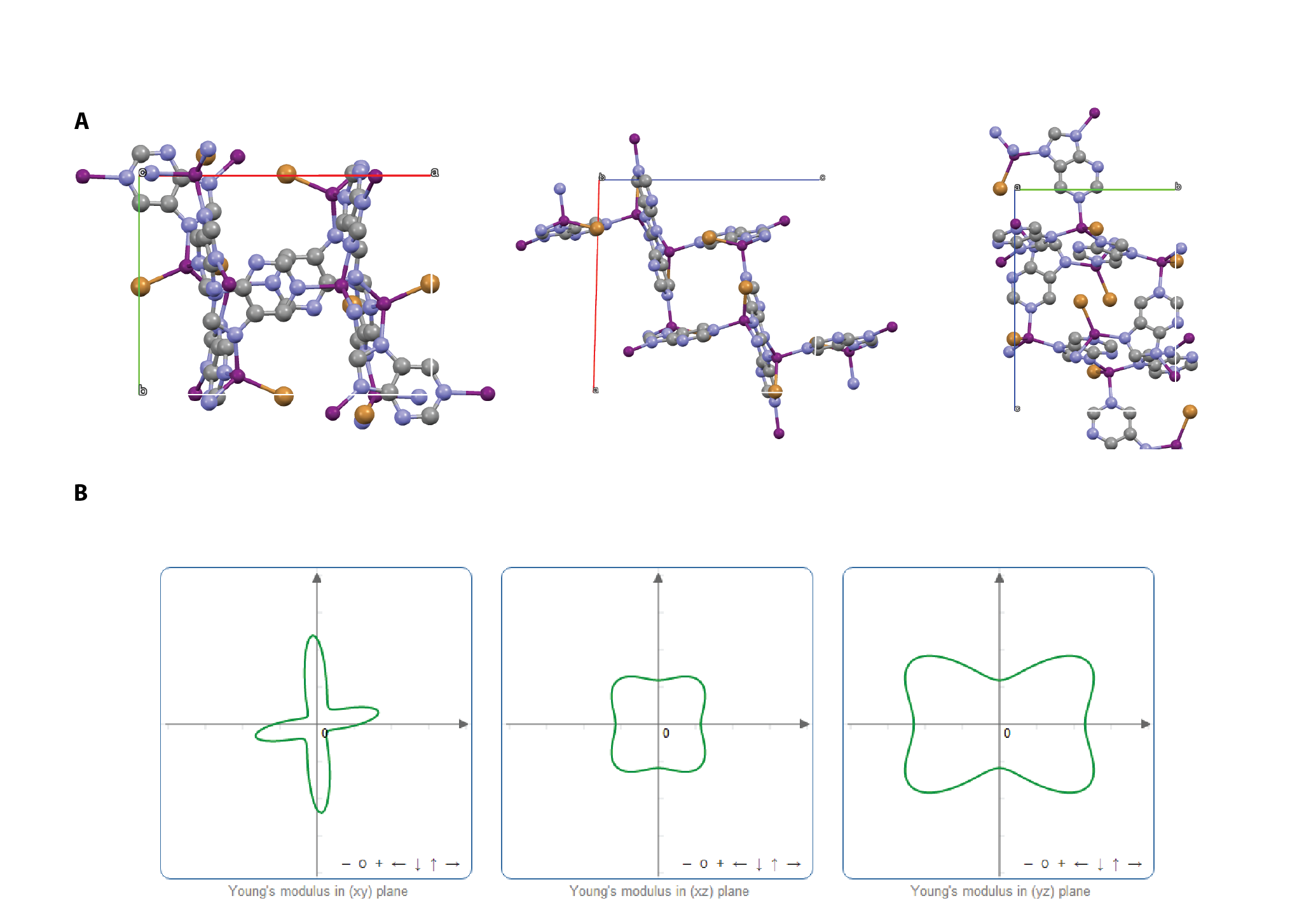


**Figure S4.** (a) Two dimensional slices through the computationally derived iso-surface of the Young’s Moduli in the ZnPurBr-ht structure. Left: xy plane, center: xz plane, right: yz plane. Bottom: Unit cells of the LT ZnPurBr structure in the corresponding planes. Purple: Zinc, Blue: Nitrogen, Grey: Carbon, Orange: Bromine. Hydrogen atoms omitted for clarity.

**Crystallographic Details**

|  | ZnPurBr-ht |
| --- | --- |
| Determined Chemical Composition  Formula weight of unit cell | ZnPurBr  264.39 |
| Calculated density gcm^-3^ | 2.271 |
| Space group | *P* 2_1_2_1_2_1_ |
| a / Å | 7.6188(15) |
| b / Å | 10.0699(7) |
| c / Å | 10.0802(14) |
| α | 90 |
| β | 90 |
| γ | 90 |
| V / Å**^3^** | 773.36(19) |
| Z | 4 |
| Crystal morphology | prism |
| Temperature / K | 298.2(7) |
| Radiation Type | Mo Kα |
| Wavelength / Å | 0.71073 |
| Unique / Total reflections | 763/12865 |
| Residual e- density min/max | -1.06/0.95 |
| RF | 0.2124 |
| RF[I>2σ(I)] | 0.2083 |

**References**

1. Kahr, J., et al., *Synthetic control of framework zinc purinate crystallisation and properties of a large pore, decorated, mixed-linker RHO-type ZIF.* Chemical Communications, 2012. **48**(53): p. 6690-6692.

2. Macrae, C.F., et al., *Mercury CSD 2.0 - new features for the visualization and investigation of crystal structures.* Journal of Applied Crystallography, 2008. **41**: p. 466-470.

3. Oliver, W.C. and G.M. Pharr, *Measurement of hardness and elastic modulus by instrumented indentation: Advances in understanding and refinements to methodology.* Journal of Materials Research, 2004. **19**(1): p. 3-20.

4. Tan, J.C., T.D. Bennett, and A.K. Cheetham, *Chemical structure, network topology, and porosity effects on the mechanical properties of Zeolitic Imidazolate Frameworks.* Proceedings of the National Academy of Sciences of the United States of America, 2010. **107**(22): p. 9938-9943.
